# Predicting radiocephalic arteriovenous fistula success with machine learning

**DOI:** 10.1038/s41746-022-00710-w

**Published:** 2022-10-25

**Authors:** Patrick Heindel, Tanujit Dey, Jessica D. Feliz, Dirk M. Hentschel, Deepak L. Bhatt, Mohammed Al-Omran, Michael Belkin, C. Keith Ozaki, Mohamad A. Hussain

**Affiliations:** 1 Division of Vascular and Endovascular Surgery, Department of Surgery, Brigham and Women’s Hospital, Harvard Medical School, Boston, MA USA; 2 Center for Surgery and Public Health, Brigham and Women’s Hospital, Boston, MA USA; 3 Division of Renal Medicine, Department of Medicine, Brigham and Women’s Hospital, Harvard Medical School, Boston, MA USA; 4 Division of Cardiovascular Medicine, Department of Medicine, Brigham and Women’s Hospital, Harvard Medical School, Boston, MA USA; 5 Division of Vascular Surgery and Li Ka Shing Knowledge Institute, St. Michael’s Hospital, University of Toronto, Toronto, ON Canada; 6 Department of Surgery, King Faisal Specialist Hospital and Research Center, Riyadh, Saudi Arabia

**Keywords:** Medical research, Prognosis, End-stage renal disease, Vascular diseases

## Abstract

After creation of a new arteriovenous fistula (AVF), assessment of readiness for use is an important clinical task. Accurate prediction of successful use is challenging, and augmentation of the physical exam with ultrasound has become routine. Herein, we propose a point-of-care tool based on machine learning to enhance prediction of successful unassisted radiocephalic arteriovenous fistula (AVF) use. Our analysis includes pooled patient-level data from 704 patients undergoing new radiocephalic AVF creation, eligible for hemodialysis, and enrolled in the 2014–2019 international multicenter PATENCY-1 or PATENCY-2 randomized controlled trials. The primary outcome being predicted is successful unassisted AVF use within 1-year, defined as 2-needle cannulation for hemodialysis for ≥90 days without preceding intervention. Logistic, penalized logistic (lasso and elastic net), decision tree, random forest, and boosted tree classification models were built with a training, tuning, and testing paradigm using a combination of baseline clinical characteristics and 4–6 week ultrasound parameters. Performance assessment includes receiver operating characteristic curves, precision-recall curves, calibration plots, and decision curves. All modeling approaches except the decision tree have similar discrimination performance and comparable net-benefit (area under the ROC curve 0.78–0.81, accuracy 69.1–73.6%). Model performance is superior to Kidney Disease Outcome Quality Initiative and University of Alabama at Birmingham ultrasound threshold criteria. The lasso model is presented as the final model due to its parsimony, retaining only 3 covariates: larger outflow vein diameter, higher flow volume, and absence of >50% luminal stenosis. A point-of-care online calculator is deployed to facilitate AVF assessment in the clinic.

## Introduction

Functional vascular access is necessary for hundreds of thousands of patients in the United States living with end stage kidney disease (ESKD) and undergoing chronic intermittent hemodialysis^1^. Organized efforts to promote autogenous hemodialysis access, most notably the National Kidney Foundation’s Kidney Disease Outcomes Quality Initiative (KDOQI), have resulted in a shift away from prosthetic accesses and tunneled central venous catheters (CVCs) toward arteriovenous fistulae (AVF)^2,3^. Despite efforts to promote the utilization of autogenous access, nearly half of AVFs created are never used successfully, and 80% of patients initiate hemodialysis with a CVC^4–7^. Underlying the considerable variation in successful AVF use is uncertainty in determining access readiness for use. Newly created AVFs require a period of maturation, where vessel remodeling results in a durable cannulation segment that can be used for hemodialysis^8^. Ideally, the maturation process takes place over about 6 weeks. Some AVFs will require additional maturation time or interventions prior to use due to slow or maladaptive remodeling (e.g., intimal hyperplasia), while others may be used successfully without intervention. Significant experience is needed to determine access readiness with physical exam, yet the availability of skilled providers is highly variable^9,10^. Uncertainty about access readiness may lead to prolonged dependence on CVCs and either unnecessary or delayed interventions.

All members of the ESKD care team, including primary care doctors, nephrologists, surgeons, nurses, and technicians, should be empowered to evaluate the health of hemodialysis accesses. To that end, rules for determining hemodialysis access readiness for use have been developed using ultrasound as a relatively inexpensive, noninvasive, and simple tool to benchmark maturation progress. Existing rules are based on static thresholds which place patients into two categories: ready for use and not yet ready for use. The commonly used existing thresholds include the prior KDOQI criteria (≥600 mL/min flow volume, ≥6 mm diameter, and ≤6 mm deep to skin) and the University of Alabama at Birmingham criteria (UAB; ≥ 500 mL/min flow volume and ≥4 mm diameter)^9,11^. The current work is motivated by recognition that substantial information about access maturation is lost by dichotomizing the outcome of readiness for use with static criteria. Additionally, the existing criteria were developed and validated in smaller heterogenous cohorts with a minority of forearm accesses, making their application to the radiocephalic AVF unclear^5,9^. Statistical models for prediction of AVF use have been developed by the Hemodialysis Fistula Maturation (HFM) study investigators, but the HFM observational cohort contained a minority of forearm accesses^5^. Herein, we describe the development of a new tool for the prediction of successful radiocephalic AVF use which allows for a more nuanced clinical interpretation of access readiness with improved prediction performance when compared to both the UAB and KDOQI ultrasound threshold criteria.

## Results

### Summary statistics

**Fig. 1:**
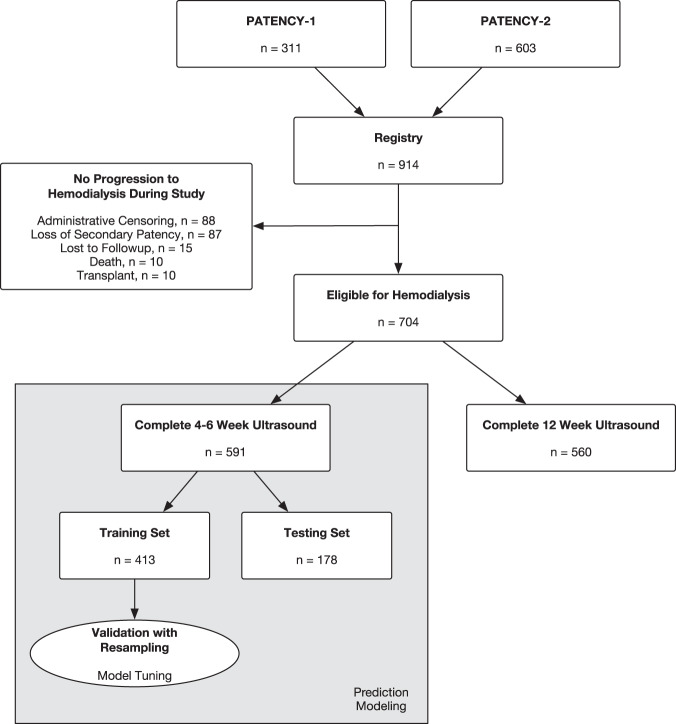
Cohort selection flowchart. Depicts flow from PATENCY-1 and PATENCY-2 randomized trials into prospective registry, eligibility for hemodialysis, receipt of postoperative ultrasound, and ultimate entry into predictive modeling cohort.

The model-building cohort of those eligible for hemodialysis during the study follow-up and with complete 4–6 week ultrasound data comprised 591 patients (Fig. 1), 55% of whom were on hemodialysis at the time of AVF creation. The mean age was 57 (SD 13) years, 22% were female, and 65% were white (Table 1). Radiocephalic AVFs were created at the wrist (75.3%), proximal forearm (22.3%), or anatomic snuffbox (2.4%). The mean intraoperative vein diameter was 3.37 mm (SD 0.82) and the mean artery diameter was 2.75 mm (SD 0.67). Patients with complete 4–6 week ultrasound data in the model-building cohort shared a similar covariate profile with the overall cohort (*n* = 914, Table 1). Median follow-up in the model-building cohort was 719 days (IQR 458–1068).

**Table 1:** Characteristics of all trial participants and prediction model-building cohort.

Baseline Characteristics	All Trial Participants	Predictive Modeling
	*N* = 914	*N* = 591
Age (Years)	57 (13)	57 (13)
Sex (Female)	203 (22%)	130 (22%)
Race		
White	619 (68%)	382 (65%)
African American	219 (24%)	149 (25%)
Other	76 (8.3%)	60 (10%)
Hispanic	156 (17%)	99 (17%)
BMI	31 [26,37]	31 [26,37]
Smoking Status		
Current	131 (14%)	91 (15%)
Former	403 (44%)	257 (43%)
Never	380 (42%)	243 (41%)
Medical History		
Diabetes	580 (63%)	382 (65%)
Hypertension	885 (97%)	573 (97%)
Heart Failure	252 (28%)	166 (28%)
Coronary Artery Disease	260 (28%)	172 (29%)
Peripheral Artery Disease	95 (10%)	47 (8.0%)
Cerebrovascular Disease	129 (14%)	79 (13%)
Any Antithrombotic	499 (55%)	336 (57%)
Statin Use	499 (55%)	312 (53%)
Prior Renal Transplant	37 (4.0%)	26 (4.4%)
Prevalent HD	409 (45%)	326 (55%)
Duration of Prior HD (Months)	9 (5,19)	9 (5,19)
Current or Prior CVC	444 (49%)	343 (58%)
Location of AVF		
Wrist	669 (73%)	445 (75%)
Forearm	220 (24%)	132 (22%)
Snuffbox	25 (2.7%)	14 (2.4%)
Intraop. Vein Diameter		
≥ 4.0 mm	282 (31%)	186 (31%)
3.0–3.9 mm	451 (49%)	296 (50%)
< 3.0 mm	181 (20%)	109 (18%)
Intraop. Artery Diameter		
≥ 3.0 mm	433 (48%)	274 (47%)
2.0–2.9 mm	450 (49%)	293 (50%)
< 2.0 mm	28 (3.1%)	22 (3.7%)
Anesthesia		
General/Local	205 (22%)	124 (21%)
Regional	709 (78%)	467 (79%)
Site Enrollment Volume		
Lower (≤ 20)	316 (35%)	197 (33%)
Mid (21–49)	303 (33%)	181 (31%)
Upper (≥ 50)	295 (32%)	213 (36%)

Data are presented as mean (standard deviation), count (percentage), and median [interquartile range].

### Ultrasound parameters

**Fig. 2:**
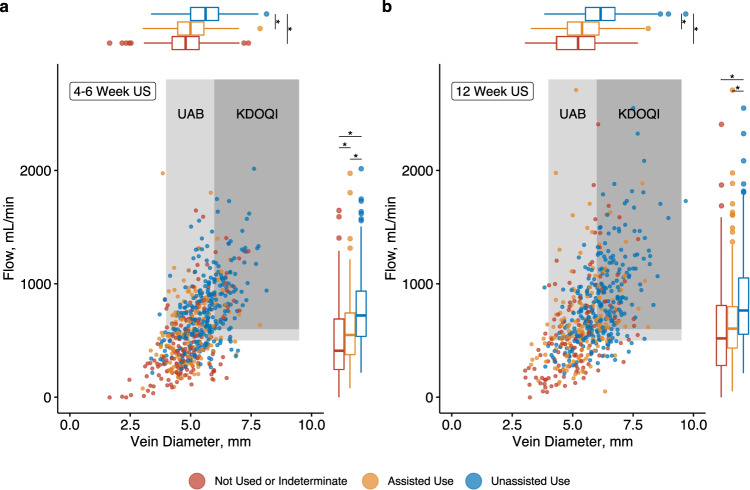
Ultrasound parameters stratified by arteriovenous fistula (AVF) use at 1-year. Points represent individual patients’ values. Data presented for 4 to 6 week **a** and 12 week **b** ultrasounds. Box plots depict median (center line), interquartile range (box bounds, 25^th^ and 75^th^ percentiles), largest value no further than 1.5 times the interquartile range (whiskers), and outlying values more than 1.5 times the interquartile range plotted individually. Colors represent AVF use category (red: not used or indeterminate, orange: assisted use, blue: unassisted use). Shaded areas correspond to flow and diameter thresholds for UAB (≥500 mL/min and ≥4 mm, light gray) and KDOQI (≥600 mL/min and ≥6 mm, dark gray) ultrasound criteria. Results of post hoc testing comparing flow and diameter between groups (Tukey’s test) are represented by asterisks (*p < 0.05).

A total of 591 patients and 560 patients had complete ultrasound data at 4–6 weeks and 12 weeks from the index surgery, respectively (Fig. 1, Table 2). Among the model-building cohort, 277 patients (46.8%) achieved unassisted AVF use within 1 year. Flow volume was lowest in those without AVF use and highest in those with unassisted AVF use at both the 4–6 week ultrasound (mean difference 250 mL/min, 95% confidence interval [CI] 175–326, Fig. 2a) and 12 week ultrasound (mean difference 235 mL/min, 95% CI 136–334, Fig. 2b). Cephalic vein diameter was smallest in those without AVF use and largest in those with unassisted AVF use at both 4–6 weeks (mean difference 0.69 mm, 95% confidence interval [CI] 0.48–0.90, Fig. 2a) and 12 weeks (mean difference 0.95 mm, 95% CI 0.70–1.2, Fig. 2b). Flow volume (mean difference 67.8 mL/min, 95% CI 39.5–96.1) and vein diameter (mean difference 0.43 mm, 95% CI 0.37–0.49) both increased between the 4–6 week and 12-week ultrasounds. Patients with successful unassisted AVF use were more likely to meet UAB (χ^2^[2] = 53.0, *p* < 0.001, Pearson’s Chi-squared test) and KDOQI (χ^2^[2] = 31.6, *p* < 0.001, Pearson’s Chi-squared test) criteria at their 4–6 week ultrasound. Among those with successful unassisted AVF use within 1-year, 73% and 52% did not meet KDOQI criteria at 4–6 weeks and 12 weeks, respectively.

**Table 2:** Successful radiocephalic arteriovenous fistula use at 1-year by ultrasound parameters at 4–6 weeks and 12 weeks.

	4–6 Week US			12 Week US		
Characteristic	Not used or indeterminate *N* = 149	Assisted use *N* = 165	Unassisted use *N* = 277	Not used or indeterminate *N* = 125	Assisted use *N* = 164	Unassisted use *N* = 271
Flow (mL/min)	515 (334)	592 (295)	766 (316)	607 (418)	666 (379)	841 (383)
Diameter (mm)	4.91 (0.96)	5.06 (0.79)	5.59 (0.89)	5.21 (1.10)	5.43 (0.93)	6.16 (0.98)
Stenosis	64 (43%)	64 (39%)	39 (14%)	43 (34%)	52 (32%)	54 (20%)
UAB Criteria Met	64 (43%)	90 (55%)	213 (77%)	66 (53%)	110 (67%)	224 (83%)
KDOQI Criteria Met	17 (11%)	13 (7.9%)	75 (27%)	21 (17%)	36 (22%)	129 (48%)

Data are presented as mean (standard deviation) and count (percentage).

### Model Performance

**Fig. 3:**
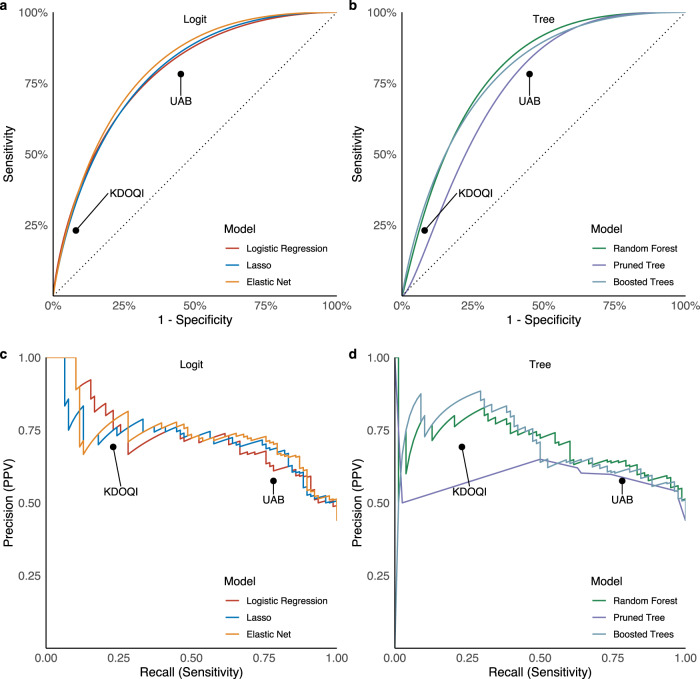
Model discrimination performance. Receiver operating characteristic curves **a**, **b** and precision-recall curves **c**, **d** depicting model performance on testing data after hyperparameter tuning and training. Panels are stratified by model type, either logit **a**, **c** or tree **b**, **d** for visual clarity. Colors identify the specific model (red: logistic regression, blue: lasso, orange: elastic net, green: random forest, purple: pruned classification tree, light blue: boosted trees). Performance of University of Alabama at Birmingham (UAB; ≥ 500 mL/min and ≥4 mm) and Kidney Disease Outcomes Quality Initiative (KDOQI; ≥ 600 mL/min and ≥6 mm) ultrasound threshold criteria also displayed as labeled points.

Discrimination performance of models based on 4–6 week ultrasound measurements and baseline characteristics in predicting 1-year unassisted AVF use are shown in Fig. 3 and Table 3. The performance of UAB and KDOQI criteria approximations based on flow and diameter thresholds were also assessed. The Lasso model (AUROC 0.794, AUPRC 0.719, accuracy 72.5%) performed nearly as well as the elastic net (AUROC 0.807, AUPRC 0.737, accuracy 71.3%) in discriminating unassisted use at one year with a much more parsimonious model (Table 3). The discrimination performance of all models was superior to that of KDOQI and UAB flow and diameter thresholds in predicting 1-year unassisted AVF use, and the models demonstrated more balanced sensitivity and specificity. The Lasso model had slightly decreased calibration when compared to the simple logistic regression model, but all models except the pruned tree demonstrated acceptable calibration (Supplementary Fig. 1). Except for the pruned tree, all models had a higher net-benefit than either the no-information strategies (use all and use none) or the existing static threshold strategies (UAB and KDOQI) across all reasonable threshold probabilities (Supplementary Fig. 2). Additional model details are included in the supplementary materials (Supplementary Table 1, Supplementary Figs. 3–6).

**Table 3:** Comparison of model performance in the hold-out testing dataset.

Model	AUROC	AUPRC	Accuracy	Sensitivity	Specificity	PPV	NPV
Elastic Net	0.807	0.737	71.3%	56.4%	83.0%	72.1%	70.9%
Lasso	0.794	0.719	72.5%	66.7%	77.0%	69.3%	74.8%
Random Forest	0.791	0.699	69.1%	66.7%	71.0%	64.2%	73.2%
Logistic Regression	0.786	0.729	73.6%	66.7%	79.0%	71.2%	75.2%
Boosted Trees	0.779	0.697	70.2%	67.9%	72.0%	65.4%	74.2%
Pruned Tree	0.730	0.586	66.9%	62.8%	70.0%	62.0%	70.7%
UAB	—	—	65.2%	78.2%	55.0%	57.5%	76.4%
KDOQI	—	—	61.8%	23.1%	92.0%	69.2%	60.5%

Except AUROC (area under the receiver operating characteristic curve) and AUPRC (area under the precision-recall curve), all metrics were calculated using a classification threshold of 0.5. *PPV* positive predictive value, *PPV* negative predictive value, *UAB* University of Alabama flow and diameter thresholds (≥500 mL/min and ≥4 mm). *KDOQI* Kidney Disease Outcomes Quality Initiative flow and diameter thresholds (≥600 mL/min and ≥6 mm).

### Final prediction model

The Lasso logistic regression model was favored as the optimal prediction model due to its combination of performance and parsimony. The final model retained only three covariates: larger outflow vein diameter in US (per mm, odds ratio [OR] 1.95, 95% CI 1.48–2.60), higher flow volume in US (per 100 mL/min, OR 1.08, 95% CI 1.00–1.17), and absence of >50% luminal stenosis on US (OR 2.74, 95% CI 1.65–4.60). No preoperative or intraoperative characteristics were retained by the Lasso. The importance of the three ultrasound variables was shared across many modeling approaches, with vein diameter and flow volume contributing more to prediction performance than all other variables in the elastic net, pruned tree, random forest, and boosted tree models (Supplementary Fig. 3). Predicted probabilities of successful unassisted AVF use by 1-year for an individual patient can be calculated using the cross-table (Extended Data Supplementary Fig. 4), the nomogram (Supplementary Fig. 5), or the online calculator application (https://patrickheindel.shinyapps.io/predict-avf/).

## Discussion

We applied machine learning methods to prospectively collected data from rigorously conducted randomized clinical trials to develop a practical tool for estimating the probability of successful unassisted radiocephalic AVF use. Our tool incorporates information from standardized postoperative duplex ultrasounds and 23 baseline clinical variables in a cohort of 591 patients with newly created radiocephalic AVFs. The final prediction model retained only three predictors (AVF flow volume, vein diameter, and ≥ 50% stenosis measured at 4–6 weeks postoperatively using duplex ultrasound) and exceeded the performance of both the UAB and KDOQI ultrasound criteria. Additionally, our model outputs predicted probabilities rather than strict class predictions (e.g., use/non-use), allowing for a more nuanced interpretation of the output.

Prediction of successful AVF use is done routinely in the clinic with physical exam, but accuracy depends on substantial skill and experience, with even the most experienced clinicians achieving an accuracy of about 80%^9,10^. Often, ultrasound is used to supplement physical exam, and existing threshold criteria guide the assessment of AVF readiness for use. Making the correct assessment has important implications for decisions regarding duration of CVC use, surgical or endovascular interventions, timing of hemodialysis initiation, and access patency—all of which contribute collectively to ESKD-related morbidity and mortality. Improving prediction of successful unassisted AVF use by supplementing clinical assessments with point-of-care estimates based duplex ultrasonography should, therefore, elevate the quality of ESKD care. The development of the UAB and KDOQI criteria have together contributed substantially to ESKD patient care and inspired the present work. Recent advances in prediction methodology, combined with the availability of high-quality granular data representing a challenging study population, motivated our development of an updated approach to AVF assessment.

After tuning, fitting, and evaluating numerous models, the Lasso was chosen as our final model. The Lasso model is appealing due to its combination of simplicity and performance, with discrimination and calibration comparable to more complex methods like random forest, and a net-benefit exceeding both UAB and KDOQI across a wide range of thresholds. Discrimination refers to the ability of a model to correctly differentiate between cases and non-cases. AUROC is a metric that can be used to quantify the overall discrimination performance of a model, and discrimination performance over a range of possible classification thresholds (Fig. 3a, b). A classification threshold is the predicted probability that, when exceeded, one would label an observation as a case. When assessing metrics like sensitivity, specificity, and positive predictive value, we chose a classification threshold of 0.5, a common default for binary classification. Other classification thresholds could be chosen by a clinician depending on the clinical need and preference for prioritization of either sensitivity or specificity.

To permit valid predictions across a range of classification thresholds, the estimated predicted probability must reflect the true probability of the outcome in the population across all possible probabilities—this property is called calibration. Calibration can be assessed visually by plotting deciles of predicted probabilities against the true proportion within that decile (Supplementary Fig. 1). A numeric assessment of calibration can be made by calculating the slope and intercept of a model regressing the outcome on the predicted log-odds of the outcome, with the perfect model having slope = 1 and intercept = 0.

The choice of classification threshold is analogous to the choice between using either the KDOQI or UAB criteria—the KDOQI criteria might be used when favoring high specificity at the expense of sensitivity, while the UAB criteria reflects a prioritization of sensitivity over specificity. Regardless, the model-based approach has a higher net-benefit across a wide range of plausible classification thresholds than either KDOQI or UAB (Supplementary Fig. 2). In decision curve analysis, net-benefit represents a summary of number of true positives and false positives and is useful in summarizing both discrimination and calibration across a range of thresholds^12^. The strategy with the highest net benefit across a plausible range of threshold values will be the optimal choice for balancing true positives and false positives. Because applying this model requires no additional information, cost, or testing than what would be necessary for either the UAB or KDOQI strategies, the model should be preferred to these static criteria regardless of the clinician’s threshold preference.

Our work confirms and extends the findings of prior studies, perhaps most notably those of the HFM study, a multi-institution prospective observational cohort study concerned with better understanding AVF maturation^13^. Prior HFM work using a backward elimination algorithm found that AVF flow volume, vein diameter, and depth from skin were the most important predictors of successful AVF use in a mixed cohort including forearm (22.7%) and upper arm (77.3%) AVFs^5^. A goal of our study was to see if the addition of expanded baseline clinical characteristics with more granular detail would enhance predictive performance in a variety of modeling approaches. Although the statistical methods employed by the HFM investigators differ in their details, our results appear to replicate and confirm the findings of the HFM study. Complex modeling strategies did not substantially improve the performance of more parsimonious approaches, and ultimately, AVF flow volume and diameter remained the most important predictors of successful AVF use.

This study has some key strengths which should be highlighted. The source data has very low missingness, high internal validity, and likely very low misclassification of predictors and outcomes due to the prospective nature of data collection for the purposes of research. Additionally, the sample is homogenous with respect to access configuration, with all participants undergoing new creation of a radiocephalic AVF. Restriction to only radiocephalic AVF eliminates any variability which may be due to inherent differences between access configurations - radiocephalic AVFs tend to be distal, smaller, and with lower flow volumes then brachiocephalic AVFs, for example. As noted above, our model’s performance exceeds that of the static criteria without requiring additional testing. The model (PREDICT-AVF) is easily accessible and practical for point-of-care applications through use of the online calculator, cross-table, or nomogram chart (https://patrickheindel.shinyapps.io/predict-avf/, Supplementary Figs. 4-5).

Our work must be interpreted with caution in the context of the study design and inherent limitations. No underlying causal framework guided our analysis, which was purely concerned with prediction. Readers should be careful to avoid making causal interpretations or attributing excess meaning to the results of individual components of any prediction model. Additionally, certain simplifying assumptions were made to assist in the construction and interpretation of the models which should be kept in mind. Competing events like loss to follow-up, death, and renal transplantation were treated as non-events in this analysis. The implication is that our model predicts the probability of being observed to have successful AVF use, rather than AVF use itself. Unfortunately, the trials did not include the collection of AVF depth information in the ultrasound protocol, and although this parameter is part of the prior KDOQI “Rule of 6 s,” we had to approximate the traditional KDOQI criteria with only flow volume and diameter. Because all accesses in this study are radiocephalic, depth seems unlikely to play as significant role in access readiness for use, and only 2.9% of patients in either study required a superficialization procedure. In addition, the PATENCY trials were conducted in North America—caution should be exercised when applying this model to patients in other settings, as AV access cannulation practices vary significantly around the world.

Finally, although the predicted probability of successful AVF use is of interest to clinicians, the implications for how to use this information to guide practice are still unclear and warrant additional investigation. For example, a clinician who sees a patient with a predicted probability of successful AVF use of 30% may choose to obtain additional imaging, intervene with a surgical or endovascular procedure to assist with maturation, abandon the AVF, or simply wait and allow more maturation time—we can make no claims about which of these strategies is optimal based on the current study. Any strategy choice needs to be the result of shared decision-making with the individualized ESKD life-plan in mind^2^.

The present study contributes to ongoing work using machine learning techniques to improve ESKD care. Techniques applied in our work can be readily expanded to other access configurations and populations. Duplex ultrasound is an important non-invasive measure of AVF maturation already in routine use. Ultrasound measurements can be translated into interpretable estimates of unassisted use success through point-of-care tools developed with machine learning.

## Methods

### Data source

We conducted a post hoc analysis of pooled patient-level data from the 2014–2019 international multicenter PATENCY-1 and PATENCY-2 phase III randomized controlled trials (trial registration: ClinicalTrials.gov; NCT02110901, July 2014; and NCT02414841, August 2015). These trials prospectively tracked clinical outcomes for up to 3 years following new radiocephalic AVF creation at 31 and 39 centers, respectively, in the United States and Canada. The primary trials’ detailed methodology and results have been published previously^14–16^.

All advanced chronic kidney disease patients undergoing radiocephalic AVF creation were eligible for enrollment in the trials. Patients with a life expectancy of <6 months, active malignancy, or prior treatment with the study drug (vonapanitase, a recombinant human elastase) were excluded from the trials. Ultimately, the trial drug vonapanitase was deemed to have limited effect on the relevant clinical outcomes at one year, and further investigation of the drug for this use-case was abandoned. Participants were followed prospectively for up to three years in a pre-specified registry of clinical outcomes. Enrollment began in July 2014 and registry follow-up ended in April 2019. Key data points collected during the trial and subsequent registry follow-up included baseline comorbidities at time of trial enrollment, anatomic and case mix characteristics, subsequent surgical or endovascular interventions, and postoperative ultrasound measurements.

Routine duplex ultrasounds (US) were performed at 4 to 6 weeks and 12 weeks from AVF creation. Outflow-vein lumen diameter was measured twice at three predetermined locations in the forearm (3 cm proximal to the AVF anastomosis, mid-forearm, and immediately below the antecubital fossa) and averaged. Flow volume was estimated from three separate measurements in the same location in the cephalic vein 5 cm proximal to the AVF anastomosis. Stenosis was dichotomized as presence or absence of ≥50% luminal narrowing at any point along the entirety of the access. Access depth was not assessed. All ultrasounds were interpreted by a blinded core lab (VasCore; Boston, MA). The methods were performed in accordance with relevant guidelines and regulations, including waiver of informed consent, and approved by the Mass General Brigham human research committee Institutional Review Board for Use of previously collected trial data from PATENCY-1 and PATENCY-2 for post hoc analysis.

### Prediction Models

We sought to build upon and refine existing threshold-based ultrasound criteria for predicting AVF maturation and suitability for use. To be included in prediction modeling, patients needed to be at risk for AVF use during the study follow-up (e.g., on hemodialysis) and have complete 4- to 6-week ultrasound data. Any patients with pre-dialysis chronic kidney disease that did not progress to requiring hemodialysis during the study follow-up were excluded (Fig. 1).

### Outcome

To improve interpretability and simplify model building, the outcome for prediction modeling was dichotomized as successful unassisted AVF use within 1-year, defined as 2-needle cannulation for hemodialysis for ≥90 days without preceding intervention. Patients who did not successfully use their AVF by one year or prior to a terminal event (death, transplant, access abandonment, or loss to follow-up) were categorized as not having successful use. For patients with prevalent hemodialysis, the one-year time window started on the day of their surgery. For patients not yet receiving hemodialysis at the time of AVF creation and who did not start hemodialysis within one-year, successful use was defined as 2-needle cannulation for all prescribed hemodialysis for a consecutive 90-day period starting within 6 weeks of hemodialysis initiation. Similar approaches have been implemented in prior analyses of AVF data^5^.

### Covariate selection

Covariates were shared by all predictive modeling processes, and included age, sex, race, ethnicity, body mass index, smoking status, medical comorbidities, hemodialysis status at the time of AVF creation, CVC history, CKD etiology, baseline vein and artery diameter measured in the operating room after induction of anesthesia, AVF location, anesthesia modality, anastomotic suture technique, statin use, antithrombotic use, and enrolling site volume. Ultrasound data from the 4–6 week visit was chosen for predictive modeling because of parallels with prior work examining prediction of unassisted AVF use, clinical relevance, and the complexity of including both 4–6 week and 12-week data together in models. Ultrasound covariates included cephalic vein diameter, AVF flow volume, and the presence or absence of ≥50% luminal stenosis. Analysis was restricted to patients with complete 4–6 week ultrasound data as described above. Covariate missingness was accounted for using K-nearest neighbors imputation^17^.

### Statistical analysis

In reporting descriptive statistics, categorical variables were summarized using frequency with percentage. Continuous variables were reported as mean with standard deviation when normally distributed, and median with interquartile range otherwise. Unadjusted comparisons of ultrasound variables were made using analysis of variance (ANOVA) followed by Tukey’s test. Paired data were compared using paired t-tests. Categorical data were compared using Pearson’s Chi-squared tests. A two-tailed alpha level of 0.05 was used. All analysis was performed using R version 4.0.5 (https://cran.r-project.org/) and the packages *tidyverse*, *tidymodels*, *glmnet, rpart*, and *ranger*.

### Modeling overview

To achieve our goal of building a predictive classification model, we explored several modeling procedures each with its own potential benefits and drawbacks. Modeling methods included traditional logistic regression, penalized logistic regression using Lasso, classification, and regression tree (CART) methods, and two ensemble classification methods: random forest and XGBoost. Each approach is distinct with differing potential benefits and drawbacks; we sought to balance model complexity, flexibility, and performance with interpretability and clinical usefulness.

Multivariable logistic regression is used as a “gold standard” in classification problems. With several covariates used for modeling, simple logistic regression can result in overfitting with bias in coefficient estimation that leads to a drop in performance when the model is used on external data. To address this issue, penalized regression techniques use coefficient shrinkage to reduce out-of-sample bias; Lasso is a popular technique due to its ability to shrink coefficients to zero, acting as an empiric variable selection method and leading to simpler final models^18^. Notably, the bias-variance tradeoff will always be a compromise and overfitting cannot be eliminated, but penalization and cross-validation techniques described here can mitigate overfitting (particularly in smaller datasets).

The CART procedure is another traditional procedure for classification, with the key benefit of flexibly producing a clinically interpretable decision rule, but with a drawback of having potentially unstable performance in external datasets even with pruning methods^19^. To overcome this issue, tree ensemble methods like the random forest and XGBoost have been developed with widespread adoption^20,21^. Random forest and XGboost are highly flexible and consider interactions between variables with relatively low bias. Random forest grows thousands of trees in a similar way to CART, but using random samples of both variables and records which are then averaged over to achieve a final model (a technique referred to as bootstrap aggregating, or “bagging”). Similarly, XGBoost can build thousands of trees, but additionally uses the error from each tree to reweight samples selected for each subsequent tree (referred to as gradient boosting), theoretically preferencing variables with the most predictive performance and de-emphasizing meaningless variables. Variable importance can be examined through a variety of methods, but a deeper understanding of the relationships between variables in ensemble techniques is challenging and can lead to skepticism from clinicians due to lower interpretability.

### Modeling details

All predictive modeling methods were built with a training, hyperparameter tuning, and testing paradigm using a combination of baseline clinical characteristics and 4–6 week US parameters described above. We performed a random 70/30 initial split into training and testing datasets prior to model building, diagnostics, or data cleaning. Continuous variables were preprocessed by centering (subtracting the average) and scaling (dividing by the standard deviation) their distributions prior to model fitting. A total of 5 missing values were imputed using K-nearest-neighbors methodology (BMI, *n* = 1; intraoperative vein diameter, *n* = 2; intraoperative artery diameter, *n* = 2)^17^. Models were built using the training dataset, and hyperparameters were tuned using grid search methods with nested 10-fold cross-validation within the training dataset.

Our modeling approach started with simple logistic regression including all covariates in a main-effects model. Next, a lasso penalized logistic regression model was fit to empirically select covariates most useful for prediction^18^. The regularization penalty was chosen to select the most parsimonious model within one standard error of the regularization penalty with the minimum 10-fold cross-validated mean log-loss. Lasso was used for variable selection for the refitting of a logistic regression model. Finally, an elastic net model was fit using a regular grid search with 10 levels and nested 10-fold cross-validation to tune both the regularization penalty value and the elastic net mixing parameter^22^. Variable importance was calculated as the absolute value of the scaled coefficients at the optimal regularization penalty.

A simple classification tree approach was also pursued with the hopes of improving interpretability in the case that a simple and useful decision tree could be identified^19^. The tree model was pruned by optimizing the complexity parameter and tree depth using a regular grid search with 10 levels and nested 10-fold cross-validation. Variable importance was calculated via the total Gini impurity reduction method.

A random forest classification model was built with the goal of increasing predictive performance at the cost of some interpretability. Hyperparameters tuned included the number of covariates for each attempted node split and the minimum node size. Hyperparameters were tuned with a regular grid search with 10 levels and nested 10-fold cross-validation. All random forest models were built with 1,000 trees. Variable importance was calculated via the Gini impurity reduction method^20,23^.

A boosted tree model was built using the XGBoost method with a logistic loss function^21^. Tree depth, minimum node size, the learning rate, and the minimum loss reduction required to make a further partition on a leaf node were tuned using nested 10-fold cross-validation and a maximum entropy grid search containing 100 hyperparameter configurations. Variable importance was calculated via the information gain method.

After hyperparameter tuning, the final models were re-fit on the entire training dataset. The final model performance was assessed on the prediction of the hold-out testing dataset. A classification threshold of 0.5 was used for all models. Receiver operating characteristic (ROC) curve plots, calibration plots, and decision curve plots were constructed for each modeling approach. Performance metrics of each modeling approach were calculated, including the area under the ROC curve (AUROC), area under the precision-recall curve (AUPRC), sensitivity, specificity, accuracy, and logistic calibration slope and intercept. The discriminative performance of each model was compared to the performance of static threshold criteria approximating the UAB (flow volume >500 mL/min and vein diameter >4 mm) and KDOQI (flow volume >600 mL/min and vein diameter >6 mm) ultrasound criteria. Decision curves were plotted for each possible strategy for AVF use prediction across a range of threshold probabilities^24^.

### Reporting summary

Further information on research design is available in the Nature Research Reporting Summary linked to this article.

## Supplementary information

Supplementary Information

Reporting Summary

## Data Availability

Limited deidentified data used for the analyses presented in this work (training and testing datasets) are available to qualified researchers on request, please email the corresponding author Dr. Mohamad Hussain, MD, PhD at mhussain7@bwh.harvard.edu.

## Code availability

Sample code for all data processing and analysis presented in this work are available on request, please email the corresponding author Dr. Mohamad Hussain, MD, PhD at mhussain7@bwh.harvard.edu.
