# Supplementary material for: Predicting radiocephalic arteriovenous fistula success with machine learning
Source: NPJ Digit Med. 2022 Oct 25;5:160. doi: 10.1038/s41746-022-00710-w (PMC9592575; doi:10.1038/s41746-022-00710-w)
Supplement: Supplementary file 1 — Supplementary Information [file 41746_2022_710_MOESM1_ESM.pdf]

Supplementary Figures

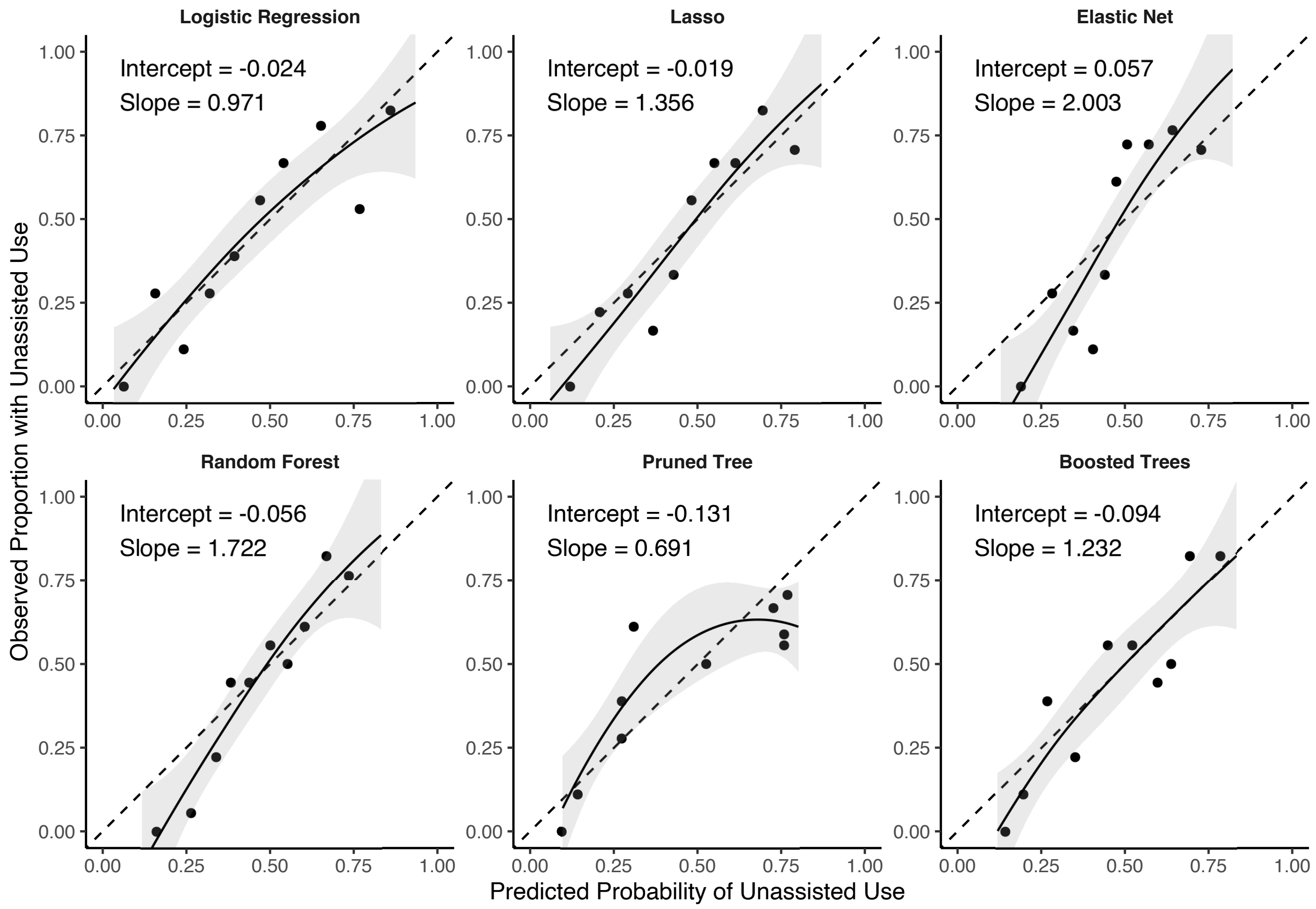

Supplementary Figure 1. Calibration plots. Calibration plots depict the observed proportion of unassisted arteriovenous fistula (AVF) use by the models' predicted probability of unassisted arteriovenous fistula use as determined by testing dataset. Logistic calibration intercept and slope are reported. Solid line represents loess nonparametric calibration curve with 95% confidence intervals. Points represent deciles of predicted probabilities. Dotted line corresponds to perfect calibration reference.

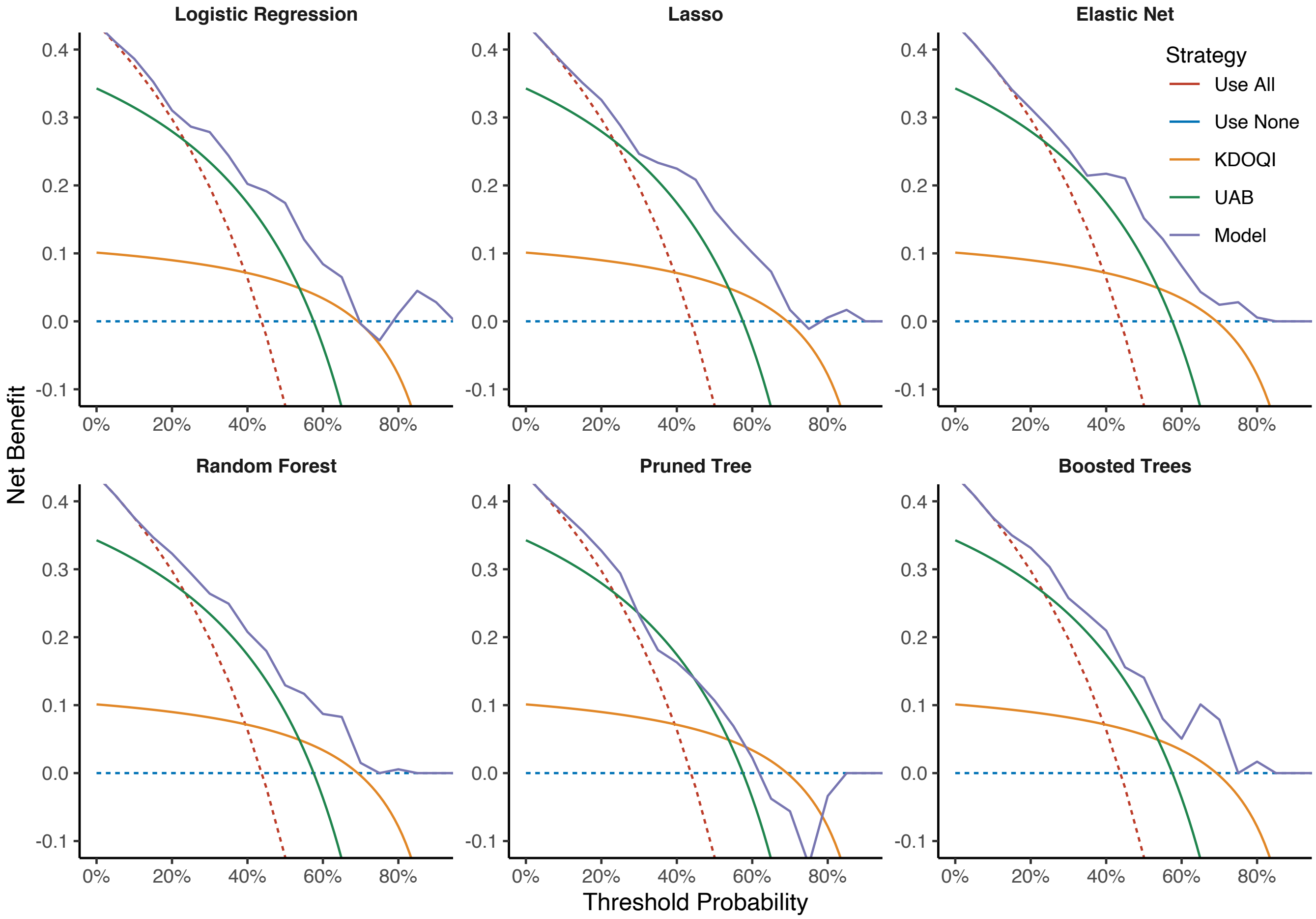

Supplementary Figure 2. Decision curves. Decision curves are plotted for each possible strategy for predicting successful unassisted AVF use at varying probability thresholds. The optimal strategy has the highest net-benefit across a range of threshold probabilities. Colors identify the strategy (dashed red: use all, dashed blue: use none, solid orange: KDOQI criteria, solid green: UAB criteria, solid purple: model).

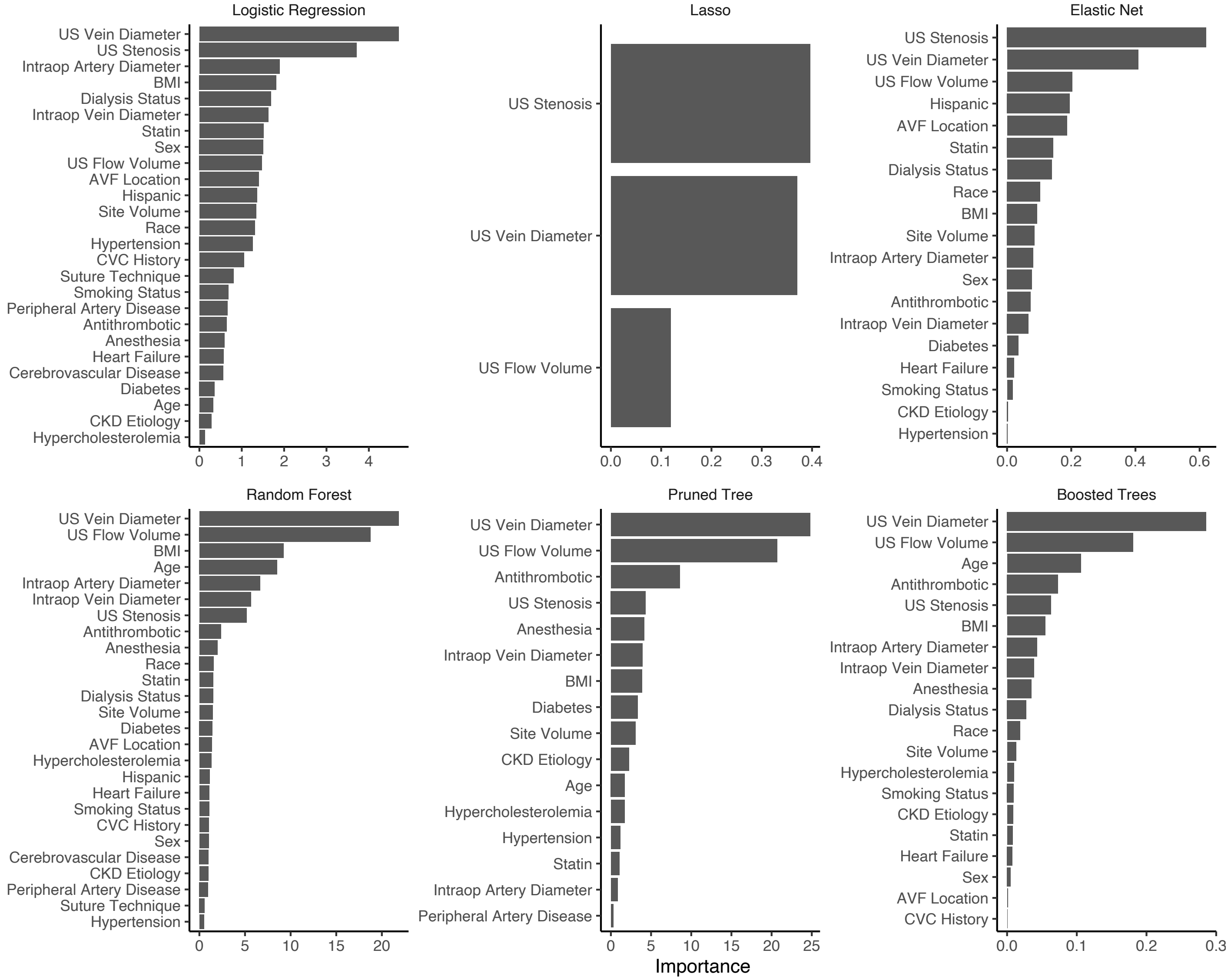

Supplementary Figure 3. Variable importance within each model. Details regarding calculation of variable importance are included in the Methods.

	No Stenosis							Stenosis						
Flow (mL/min)	2mm	3mm	4mm	5mm	6mm	7mm	8mm	2mm	3mm	4mm	5mm	6mm	7mm	8mm
200	0.08	0.15	0.26	0.41	0.57	0.72	0.84	0.03	0.06	0.11	0.20	0.33	0.49	0.65
400	0.10	0.17	0.29	0.44	0.61	0.75	0.86	0.04	0.07	0.13	0.23	0.36	0.53	0.68
600	0.11	0.20	0.32	0.48	0.64	0.78	0.87	0.04	0.08	0.15	0.25	0.40	0.56	0.72
800	0.13	0.22	0.36	0.52	0.68	0.80	0.89	0.05	0.09	0.17	0.28	0.44	0.60	0.75
1000	0.15	0.25	0.39	0.56	0.71	0.83	0.90	0.06	0.11	0.19	0.32	0.47	0.64	0.77
1200	0.17	0.28	0.43	0.60	0.74	0.85	0.92	0.07	0.12	0.22	0.35	0.51	0.67	0.80
1400	0.19	0.31	0.47	0.63	0.77	0.87	0.93	0.08	0.14	0.24	0.39	0.55	0.71	0.82
1600	0.21	0.34	0.51	0.67	0.80	0.88	0.94	0.09	0.16	0.27	0.42	0.59	0.74	0.84
1800	0.24	0.38	0.55	0.70	0.82	0.90	0.95	0.10	0.18	0.30	0.46	0.63	0.76	0.86
2000	0.27	0.42	0.58	0.73	0.84	0.91	0.95	0.12	0.21	0.34	0.50	0.66	0.79	0.88

Supplementary Figure 4. Predicted probabilities of successful unassisted AVF use by 1-year. Estimates derived from penalized logistic regression model retaining outflow vein diameter, flow volume, and presence or absence of 50% luminal stenosis. Background color corresponds to probability of successful use, with warmer colors (top left) suggesting lower probabilities.

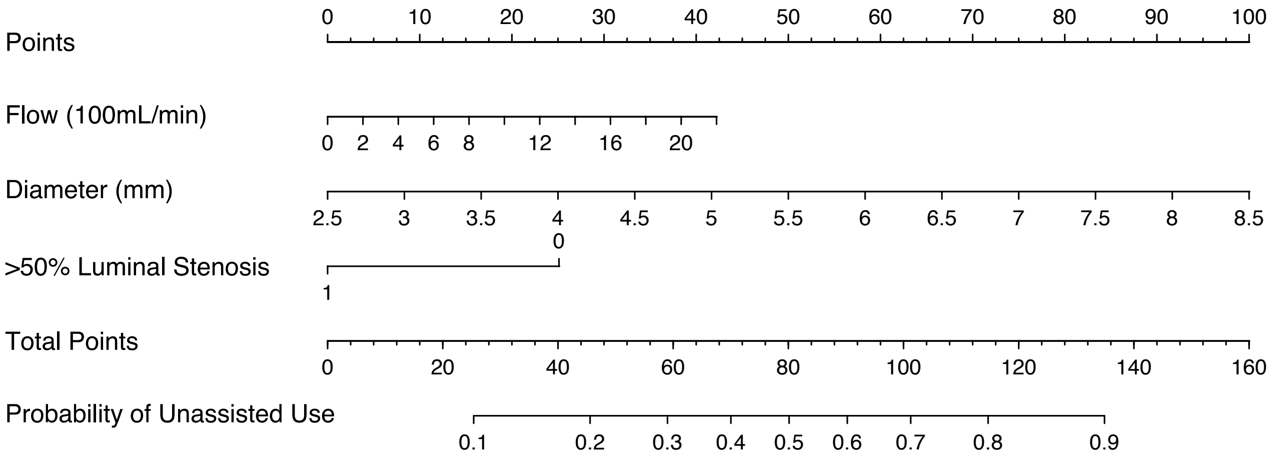

Supplementary Figure 5. Nomogram for Lasso logistic regression model. Predicted probability of 1-year unassisted radiocephalic arteriovenous fistula use can be estimated by summing points from the flow, diameter, and stenosis covariates.

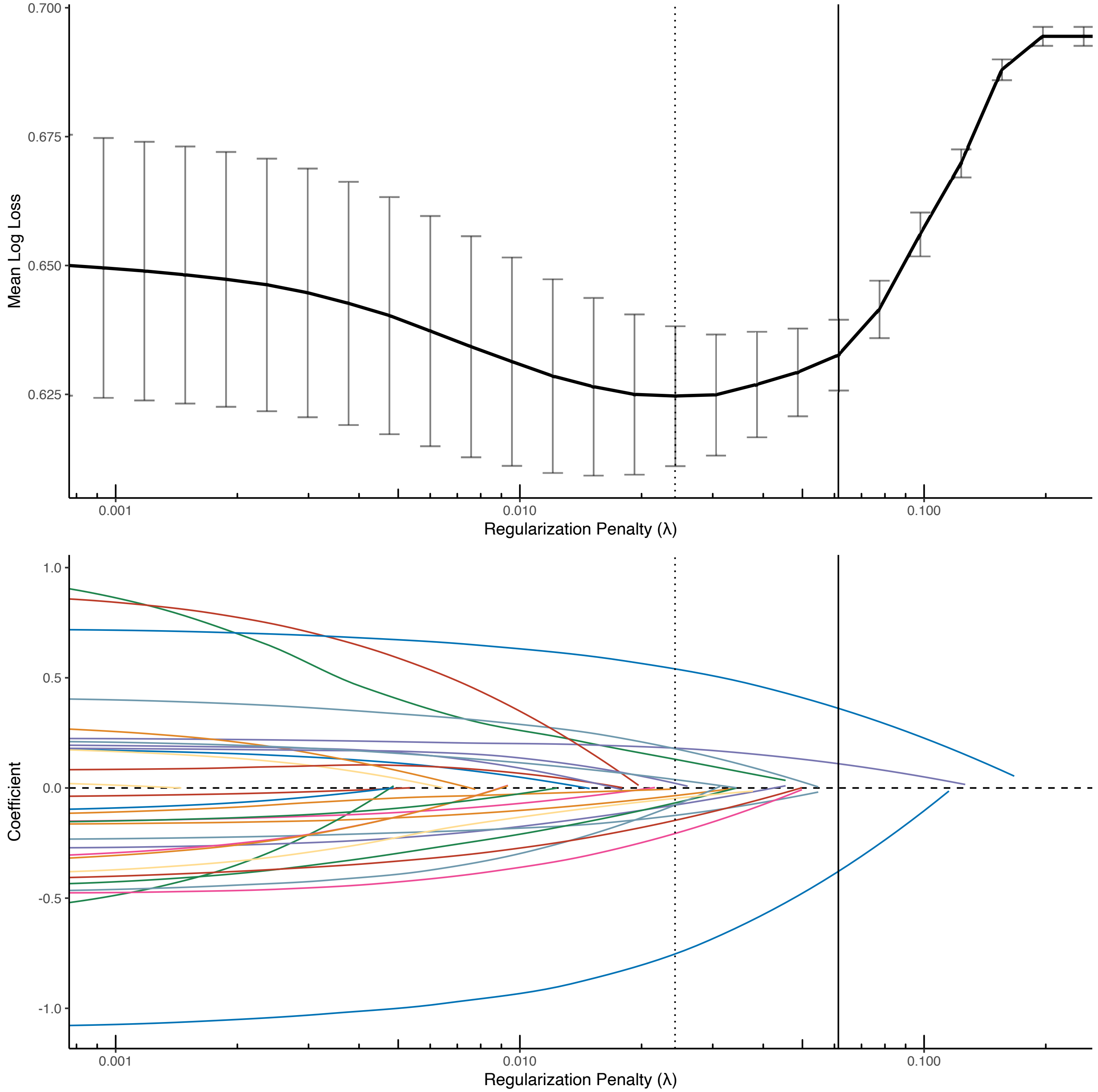

Supplementary Figure 6. Plot of the cross-validated coefficient paths and the mean log loss for the Lasso model as a function of the regularization penalty (λ). Vertical lines represent the penalty value with the minimum mean log loss (dashed) and the largest penalty value within one standard error of the minimum mean log loss (solid, selected for final model fit). Error bars represent standard-error of the mean log-loss.

Supplementary Tables

Covariates	Summary: Train N = 413	Summary: Test N = 178	Logistic Regression: OR	Logistic Regression: 95% CI
Age (Years)	57 (12)	59 (13)	1.00	0.98, 1.02
Sex (Female)	88 (21%)	42 (24%)	0.61	0.33, 1.13
Race				
White	269 (65%)	113 (63%)	—	—
African American	97 (23%)	52 (29%)	0.63	0.32, 1.25
Other	47 (11%)	13 (7.3%)	1.18	0.55, 2.51
Hispanic	69 (17%)	30 (17%)	0.63	0.31, 1.26
BMI	31 (26, 37)	31 (26, 36)	0.97	0.95, 1.00
Smoking Status				
Never	175 (42%)	68 (38%)	—	—
Currently	60 (15%)	31 (17%)	0.85	0.42, 1.70
Formerly	178 (43%)	79 (44%)	1.24	0.74, 2.10
Diabetes	263 (64%)	119 (67%)	0.86	0.42, 1.79
Hypertension	400 (97%)	173 (97%)	1.89	0.43, 8.67
Hypercholesterolemia	293 (71%)	118 (66%)	1.07	0.57, 2.05
Heart Failure	113 (27%)	53 (30%)	0.85	0.49, 1.46
Peripheral Artery Disease	34 (8.2%)	13 (7.3%)	1.32	0.53, 3.23
Cerebrovascular Disease	54 (13%)	25 (14%)	1.18	0.59, 2.35
Antithrombotic Use	228 (55%)	108 (61%)	0.82	0.49, 1.37
Statin Use	219 (53%)	93 (52%)	0.66	0.38, 1.15
Prevalent HD	220 (53%)	106 (60%)	2.75	0.87, 9.51
CKD Etiology				
Diabetes	196 (47%)	84 (47%)	—	—
HTN	99 (24%)	50 (28%)	1.08	0.52, 2.24
Other	118 (29%)	44 (25%)	0.85	0.39, 1.85
Current or Prior CVC	235 (57%)	108 (61%)	0.53	0.16, 1.69
Intraop. Vein Diameter (mm)	3.00 (3.00, 4.00)	3.00 (3.00, 4.00)	1.29	0.94, 1.77
Intraop. Artery Diameter (mm)	2.50 (2.40, 3.00)	2.75 (2.20, 3.00)	0.67	0.43, 1.02
Suture Technique				
Interrupted	41 (9.9%)	18 (10%)	—	—
Running	372 (90%)	160 (90%)	0.73	0.31, 1.75
AVF Location				
Wrist/Snuffbox	317 (77%)	142 (80%)	—	—
Forearm	96 (23%)	36 (20%)	1.48	0.83, 2.65
Anesthesia				
General/Local	84 (20%)	40 (22%)	—	—
Regional	329 (80%)	138 (78%)	1.22	0.66, 2.26
Site Enrollment Volume				
Lower (≤ 20)	138 (33%)	59 (33%)	—	—
Mid (21-49)	127 (31%)	54 (30%)	0.70	0.38, 1.28
Upper (≥ 50)	148 (36%)	65 (37%)	0.72	0.40, 1.31
Flow Volume (mL/min)	624 [412, 879]	574 [377, 794]	1.07	0.98, 1.17
Vein Diameter (mm)	5.30 (0.93)	5.21 (0.92)	2.17	1.58, 3.03
Luminal Stenosis	108 (26%)	59 (33%)	0.33	0.18, 0.58

Supplementary Table 1. Summary of random train and test split covariates with simple logistic regression model fit on the training data. Summary data are presented as mean (standard deviation), count (percentage), and median [interquartile range]. OR = Odds Ratio, CI = Confidence Interval. Flow volume scaled to 100mL/min for odds ratio calculation.
