# Supplementary material for: Predicting radiocephalic arteriovenous fistula success with machine learning
Source: NPJ Digit Med. 2022 Oct 25;5:160. doi: 10.1038/s41746-022-00710-w (PMC9592575; doi:10.1038/s41746-022-00710-w)
Supplement: Supplementary file 2 — Reporting Summary [file 41746_2022_710_MOESM2_ESM.pdf]

nature portfolio

Corresponding author(s): Mohamad Hussain, MD, PhD
Brigham and Women's Hospital
75 Francis Street, Boston, MA 02115

Last updated by author(s): Sep 2, 2022

Reporting Summary

Nature Portfolio wishes to improve the reproducibility of the work that we publish. This form provides structure for consistency and transparency in reporting. For further information on Nature Portfolio policies, see our Editorial Policies and the Editorial Policy Checklist.

Statistics

For all statistical analyses, confirm that the following items are present in the figure legend, table legend, main text, or Methods section.

n/a	Confirmed	
☐	☒	The exact sample size (*n*) for each experimental group/condition, given as a discrete number and unit of measurement
☐	☒	A statement on whether measurements were taken from distinct samples or whether the same sample was measured repeatedly
☐	☒	The statistical test(s) used AND whether they are one- or two-sided<br>*Only common tests should be described solely by name; describe more complex techniques in the Methods section.*
☐	☒	A description of all covariates tested
☐	☒	A description of any assumptions or corrections, such as tests of normality and adjustment for multiple comparisons
☐	☒	A full description of the statistical parameters including central tendency (e.g. means) or other basic estimates (e.g. regression coefficient) AND variation (e.g. standard deviation) or associated estimates of uncertainty (e.g. confidence intervals)
☒	☐	For null hypothesis testing, the test statistic (e.g. *F*, *t*, *r*) with confidence intervals, effect sizes, degrees of freedom and *P* value noted<br>*Give P values as exact values whenever suitable.*
☒	☐	For Bayesian analysis, information on the choice of priors and Markov chain Monte Carlo settings
☐	☒	For hierarchical and complex designs, identification of the appropriate level for tests and full reporting of outcomes
☒	☐	Estimates of effect sizes (e.g. Cohen's *d*, Pearson's *r*), indicating how they were calculated

Our web collection on statistics for biologists contains articles on many of the points above.

Software and code

Policy information about availability of computer code

Data collection	n/a
Data analysis	All analysis was performed using R version 4.0.5 (https://cran.r-project.org/) and the packages tidyverse, tidymodels, glmnet, rpart, and ranger. Sample code for all data processing and analysis presented in this work are available on request, please contact the corresponding author.

For manuscripts utilizing custom algorithms or software that are central to the research but not yet described in published literature, software must be made available to editors and reviewers. We strongly encourage code deposition in a community repository (e.g. GitHub). See the Nature Portfolio guidelines for submitting code & software for further information.

Data

Policy information about availability of data

All manuscripts must include a data availability statement. This statement should provide the following information, where applicable:

- Accession codes, unique identifiers, or web links for publicly available datasets
- A description of any restrictions on data availability
- For clinical datasets or third party data, please ensure that the statement adheres to our policy

Limited deidentified data used for the analyses presented in this work are available on request, please contact the corresponding author.

Human research participants

Policy information about studies involving human research participants and Sex and Gender in Research.

Reporting on sex and gender	Sex was included in our prediction models - biological sex is known to be associated with hemodialysis access outcomes. No sex-specific conclusions are made in our work. Only 22% of our study population were female, and this is reported clearly in the manuscript.
Population characteristics	We conducted a post hoc analysis of pooled patient-level data from the 2014-2019 international multicenter PATENCY-1 and PATENCY-2 phase III randomized controlled trials (trial registration: ClinicalTrials.gov; NCT02110901, July 2014; and NCT02414841, August 2015). All advanced chronic kidney disease patients undergoing radiocephalic AVF creation were eligible for enrollment in the trials. Patients with a life expectancy of <6 months, active malignancy, or prior treatment with the study drug (vonapanitase, a recombinant human elastase) were excluded from the trials. Mean age was 57 (SD 13) years, 22% were female, and 65% were white.
Recruitment	The trials included in our work attempted to recruit all patients eligible for new radiocephalic AVF creation at 31 and 39 centers, (PATENCY-1 and -2) in the United States and Canada from 2014-2019. Recruitment was done in hemodialysis access clinics. The recruitment is limited to the centers enrolling in the trial, which may limit the generalizability of our models. This has been acknowledged explicitly in the discussion.
Ethics oversight	The methods were performed in accordance with relevant guidelines and regulations, including waiver of informed consent, and approved by the Mass General Brigham human research committee Institutional Review Board for Use of previously collected trial data from PATENCY-1 and PATENCY-2 for post hoc analysis.

Note that full information on the approval of the study protocol must also be provided in the manuscript.

Field-specific reporting

Please select the one below that is the best fit for your research. If you are not sure, read the appropriate sections before making your selection.

☒ Life sciences ☐ Behavioural & social sciences ☐ Ecological, evolutionary & environmental sciences

For a reference copy of the document with all sections, see nature.com/documents/nr-reporting-summary-flat.pdf

Life sciences study design

All studies must disclose on these points even when the disclosure is negative.

Sample size	This was a sample of convenience from previously conducted randomized trials. The final sample size was 591 participants, which is the largest group of patients undergoing radiocephalic AVF creation to our knowledge. No sample size or power calculations were appropriate for our design.
Data exclusions	All advanced chronic kidney disease patients undergoing radiocephalic AVF creation were eligible for enrollment in the trials. Patients with a life expectancy of <6 months, active malignancy, or prior treatment with the study drug (vonapanitase, a recombinant human elastase) were excluded from the trials. Any patient with missing 6-week ultrasound data was excluded from our study, as this information was critical for model building. We acknowledge the risk of decreased generalizability given these restrictions in our manuscript.
Replication	We assessed the performance of our models using an internal validation process with crossvalidation and a training/test split. We plan to replicate our findings externally in a followup study using publicly available data from the NIH Hemodialysis Fistula Maturation study.
Randomization	n/a - not a causal inference study
Blinding	n/a - not a causal inference study

Reporting for specific materials, systems and methods

We require information from authors about some types of materials, experimental systems and methods used in many studies. Here, indicate whether each material, system or method listed is relevant to your study. If you are not sure if a list item applies to your research, read the appropriate section before selecting a response.

Materials & experimental systems

n/a	Involved in the study
☒	☐ Antibodies
☒	☐ Eukaryotic cell lines
☒	☐ Palaeontology and archaeology
☒	☐ Animals and other organisms
☐	☒ Clinical data
☒	☐ Dual use research of concern

Methods

n/a	Involved in the study
☒	☐ ChIP-seq
☒	☐ Flow cytometry
☒	☐ MRI-based neuroimaging

Clinical data

Policy information about clinical studies

All manuscripts should comply with the ICMJE guidelines for publication of clinical research and a completed CONSORT checklist must be included with all submissions.

Clinical trial registration: ClinicalTrials.Gov: NCT02110901 and NCT02414841

Study protocol: Available in original trial publications and at ClinicalTrials.Gov

Data collection: 31 (PATENCY 1) and 39 (PATENCY 2) centers in the United States and Canada from 2014-2019

Outcomes: The outcome for all prediction models was successful arteriovenous unassisted fistula use within 1-year, defined as 2-needle cannulation for hemodialysis for at least 90 concurrent days without preceding intervention. This outcome was chosen due to its clinical relevance and alignment with prior work. The TRIPOD guidelines for reporting of clinical prediction models were followed and the checklist is included in our submission
